# Serological and Molecular Surveillance of Infectious Bronchitis Virus Infection in Free-Range Chickens and Guinea Fowls in the Ga-East District of Ghana

**DOI:** 10.1155/2018/4949580

**Published:** 2018-08-06

**Authors:** Matilda Ayim-Akonor, Doreen Dela Owusu-Ntumy, Hilda Emefa Ohene-Asa, Agyekum Oduro-Abrokwa, Patricia Hammond, Michael Appenteng, Daniel Annan

**Affiliations:** Council for Scientific and Industrial Research-Animal Research Institute, P.O. Box AH 20, Achimota, Accra, Ghana

## Abstract

Infectious bronchitis is an economically important disease with worldwide distribution. Information is available on the presence of infectious bronchitis virus in commercial chicken in parts of Ghana but there is no information on free-range poultry and guinea fowls in the country. Possible IBV infections among free-range chickens and guinea fowls in Abokobi and Frafraha communities in the Ga-East district of the Greater Accra Region of Ghana were investigated using serology and PCR. Blood, tracheal, and cloacal swabs were obtained from 219 free-range chickens and guinea fowls with no respiratory symptoms and no history of IBV vaccination. Sera were evaluated for IBV antibodies by ELISA using commercial IBV test kit from IDEXX, Inc., USA. Swab samples were evaluated for S1 glycoprotein gene by one-step RT PCR. All the swab samples tested negative for IBV. 16% of all tested sera were positive for IBV. IBV seroprevalence in guinea fowls was 0%. 21.2% of sera from local chickens were positive for IBV. The seroprevalence of IBV among local chickens from Frafraha was 30% and that of Abokobi was 7.7%. This study shows exposure of local chickens in the study communities to IBV.

## 1. Introduction

Poultry production, including chickens, ducks, and guinea fowls, is an ancient agricultural activity worldwide (Njue et al., 2002; Ideris et al., 1990) [1]. The village chicken, or free-range, backyard, indigenous, local, or rural chicken, as it is often called, has been an integral part of the lives of many Africans for decades, contributing substantially to improvement in the livelihood of the people. They are found in nearly every household in rural and periurban communities [2, 3]. In Ghana, the population of the village chicken is estimated to constitute 60-80% of the national poultry population [4]. Guinea fowls also comprised seven percent of the 2009 national poultry population [5] and the population is growing. A number of government interventions and donor funding opportunities are in place to stimulate the growth of guinea fowl production [6].

The free-range guinea fowl and local chicken are raised with minimum capital input from the farmer. They scavenge for food themselves and are occasionally supplemented with cereals such as maize and millet and kitchen wastes from the household [7–10]. They have no specific shelter and often sleep on trees, in the kitchen, or on the compound and are left to the perils of the weather and predators. Where shelter is provided, the coop is often small and overcrowded [9, 11, 12].

These local birds, with the limited care, are believed to be generally hardy and resistant to various poultry diseases [13]. In spite of this belief though, literature shows that local chickens are susceptible to some of the common poultry diseases such as Newcastle, Gumboro, Coccidiosis, fowl pox, infectious coryza, chronic respiratory diseases, and internal and external parasites which account for high percentage of poultry losses annually, as high as 50-70% [4, 7, 9, 14] (Melewas, 1989; Yongolo, 1996; FASDEP, 2002, FAOSTAT, 2005).

Newcastle disease is considered an important disease of local chickens in many African countries and is often diagnosed by experience, with little or no laboratory investigations [7, 9, 14] (El-Yuguda et al., 2005). The lack of laboratory confirmation of disease in the local birds may frequently result in misdiagnosis as there are a number of other viruses such as infectious bronchitis virus (IBV) and avian metapneumovirus (aMPV) whose infections produce similar respiratory symptoms as those of Newcastle disease virus (NDV) in poultry [15].

Infectious bronchitis is an acute and highly contagious disease of the respiratory and urogenital tract of chickens. It is ubiquitous in commercial chicken producing regions and economically important worldwide (Jackwood and Wit, 2014). The respiratory form of the disease is characterized by tracheal rales, cough, sneeze, and nasal discharge and gasping. Infections of the oviduct and kidneys cause egg production losses, poor egg quality, urate deposits in kidneys, and increased mortality [16] (Cook et al., 2012). The disease is caused by a single stranded RNA Coronavirus called infectious bronchitis virus (IBV) (Cavanagh and Gelb, 2008).

The involvement of IBV in outbreaks of respiratory diseases in Ghana has been confirmed in commercial chickens in the Ga-East district [17, 18]. However, such vital information is lacking where local chickens and guinea fowls are concerned. If local chickens and guinea fowls are susceptible to some of the devastating diseases afflicting commercial poultry, will they be susceptible to IBV which has already been confirmed in the Ga-East district? Thus, we set out to determine whether local chickens and guinea fowls in the Ga-East district have been exposed to IBV infection and if so, how widespread the exposure is. This will add to ways to help improve management of respiratory diseases in the district and elsewhere in Ghana.

## 2. Materials and Methods

The study was conducted in the Ga-East district of the Greater Accra region of Ghana. Prior to sampling, households in Frafraha and Abokobi communities were visited to identify houses that had indigenous chickens and/or guinea fowls. The inclusion criteria for sampling were all indigenous chickens and guinea fowls older than 2 months in these communities. In addition, accessibility to the house, willingness of owners to participate in the study, and availability of birds at the time of sampling were also used. Samples were collected from November to December 2016.

### 2.1. Sampling

Birds were inspected upon arrival to a house and those younger than 2 months were excluded from sampling. From each bird in a household that met the criteria, three (3) types of samples were taken. Blood was drawn from the neck vein of the bird with a disposable 2 mL syringe and needle and transferred into a 4-mL plain vacutainer tube. Tubes were prelabelled and slanted on racks at an angle of about 45°C to facilitate clotting and sera separation. Using rayon swabs (Copan diagnostics, Italy), the trachea and cloaca of each bird were then swabbed separately and placed into a labelled microcentrifuge tube containing 1 mL viral transport medium (VTM). Tubes were immediately placed on ice in a cool box. Basic data of flock size, age of birds, and reasons for keeping the animals were collected from the owners by questionnaire administration. Samples were transported to the CSIR-Animal Research Institute, Frafraha, within 3 hours of collection.

### 2.2. Initial Laboratory Analysis

In the laboratory, swabs in VTM were immediately stored at −80°C until needed. Vacutainers containing clotted blood were placed on the laboratory bench for at least 1 h for further separation. Vacutainers were centrifuged at 1500 rpm for 3 mins. Sera were harvested into prelabelled 2 mL centrifuge tubes and stored at −20°C until needed.

### 2.3. Serological Analysis

Each serum sample was singly tested for IBV antibodies using the enzyme-linked immunosorbent assay (ELISA) technique. A commercial IBV Antibody Test kit from IDEXX Laboratories Inc. (USA) which has also been used to detect IBV exposure in birds other than chickens (Sabarinath et al., 2011) was used. Appropriate positive and negative controls included in the test kit were added to each plate run. The manufacturer's instructions were followed with some slight modifications. Briefly, test sera and ELISA test reagents were bought to room temperature. Prior to being assayed, a 1:500 dilution of the samples was made with manufacturer's diluent in a 2-step process. 100 *μ*l of each diluted sample was then pipetted into the appropriate well on the antigen-coated plate. One hundred microliters of undiluted positive and negative controls was added to their appropriate wells in duplicate. The plate was incubated for 30 mins at room temperature. Plates were then manually washed five times with deionised water and blotted dry on laboratory tissue paper after washing. Hundred microliters of conjugate was added to all wells and the plate was incubated at room temperature for 30±2 mins. Washing and blotting were repeated as described above. One hundred microliters of TMB substrate was added to all wells and incubated at room temperature for 15±1 mins. To stop the reaction, 100 *μ*l of stop solution was added to all the wells. The optical density (OD)/absorbance value of each sample on the test plate was measured with Varioskan Lux (Thermo Scientific) at a wavelength of 650 nm.

### 2.4. Molecular Analysis

Samples from a particular source and species were processed together. RNA was extracted from all samples using the Qiagen viral RNA mini kit and following the manufacturer's instructions. Ma5 vaccine and nuclease-free H_2_O were used as extraction positive and negative controls, respectively. Amplification of the S1 glycoprotein gene common to all IBV serotypes and the most reliable one in discriminating all IBV strains [19] was carried out using the Invitrogen Superscript III One-Step RT PCR kit. The primer pair IBV LC3 5′-ACA GAT TGC TTG CAA CCA C-3′ and LC5 5′-ACT GGC AAT TTT TTC AGA-3′ [20] which gives a PCR product of 383 bp was used. The reaction was performed in a 200 *μ*l thin walled PCR tube in a final reaction volume of 25 *μ*L in a GeneAmp® PCR System 9700 thermal cycler (Applied Biosystems). The reaction comprised 2X reaction buffer (12.5uL), 10 pmol IBV LC3 (0.5 uL), 10 pmol IBV LC5 (0.5 uL), RNasin Inhibitor (0.5 uL), nuclease-free H_2_O (5 uL), Superscript III (1 uL), and RNA extract (5 uL). Cycling conditions were reverse transcription at 50°C for 30 mins, activation step at 94°C for 2 mins, followed by 45 cycles of denaturation at 94°C for 15 sec, primer annealing at 50°C for 30 sec, extension at 68°C for 1 min, and final extension at 68°C for 5 mins. Two additional controls were added to each reaction set. These were PCR negative control (nuclease-free H_2_O) and PCR positive control (Ma5 vaccine).

### 2.5. Agarose Gel Electrophoresis

Amplicons were resolved on a 1.5% agarose gel stained with ethidium bromide and visualised with a GelDoc-It®2 Imager (UVP, USA) after 60 mins of electrophoresis in a 1% TBE buffer.

### 2.6. Data Analysis

OD values were transferred onto an excel worksheet. The Positive Control Means (PCX) and Negative Control Means (NCX) for each test plate were calculated (Microsoft Excel, Microsoft Office 15). An assay was accepted to be valid when the NCX absorbance was less than or equal to 0.150 and the difference between PCX and NCX was greater than 0.075. The relative level of IBV antibody in the sample was determined by calculating the sample to positive (S/P) ratio using the following formula:

*S*/*P* = *Sample*  (*OD*) − *NCX*  (*OD*)/(*OD*) − *NCX*  (*OD*), where S/P is sample to positive ratio, Sample (OD) is OD of test serum, NCX (OD) is mean OD of negative control, and PCX (OD) is mean OD of positive control.

Serum sample with S/P ratios less than or equal to 0.20 was considered negative; S/P ratio greater than 0.20 was considered positive. Data interpretation was as provided by the manufacturer.

Data was exported and analysed using SPSS 17.0.1 (SPSS Inc.). MS prevalence was calculated using the formula:

(1) Prevalence=Number  of  positives  detectedTotal  number  of  samples  analyzed×100%

Chi-square test was performed to analyse IBV prevalence between birds in the two (2) communities. P < 0.05 was considered significant.

## 3. Results and Discussion

**Figure 1:**
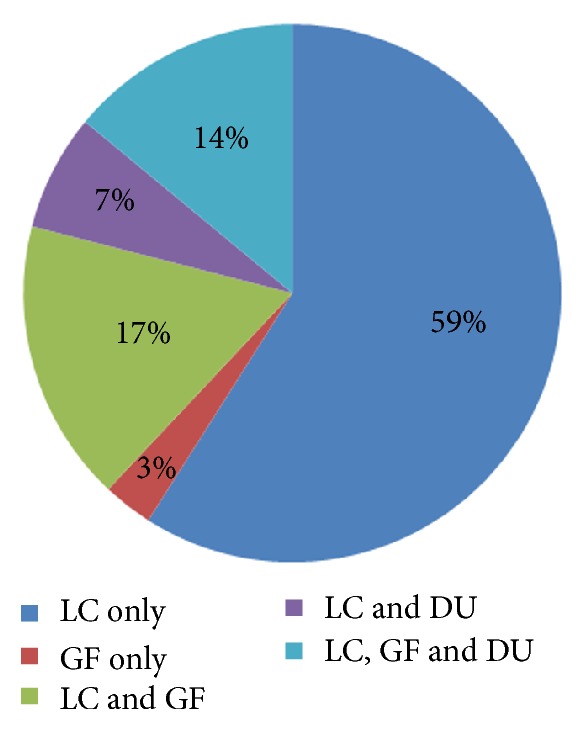
Percentage distribution of poultry species in households. LC= local chicken, GF= guinea fowl, and DU= duck.

Samples were collected from 29 households in the 2 communities during the 2-month period. All sampled poultry were free of respiratory symptoms at the time of sampling. The type and number of poultry kept by households differed. Three (3) types of poultry, local chickens, guinea fowls, and ducks, were often kept. Majority (59%) of households kept only local chickens while very few households (3%) kept only guinea fowls. 17% of households kept both local chickens and guinea fowls and 7% kept local chickens and duck. 14% households kept all three (3) species (Figure 1). The number of guinea fowls per household ranged between 5 and 20 and that of local chickens ranged from a minimum of 12 to a maximum of 28. The ages of the guinea fowls were between 4 and 8 months and those of the local chickens were between 5 months and 3 years.

In all, 219 local chickens and guinea fowls were sampled during the period. The total number of samples obtained from both communities was nearly equal (Table 1). However, there was a variation in the distribution of samples according to poultry species in the communities. Samples were collected from a total of 54 guinea fowls. Of this, more than 88% were obtained from Abokobi community and 11% from the Frafraha community (Table 1). A total of 165 local chickens were sampled from both communities. More than half (61%) of the chicken samples were obtained from the Frafraha community and 39% from Abokobi community (Table 1).

### 3.1. IBV in Guinea Fowls

**Figure 2:**
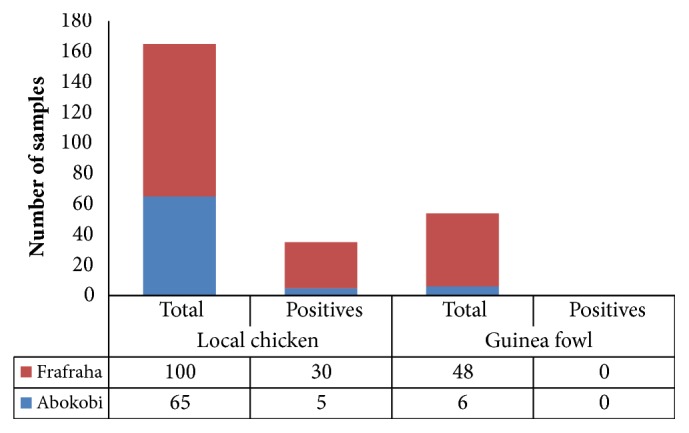
Distribution of IBV among local chickens and guinea fowls in Frafraha and Abokobi community.

None of the 54 guinea fowl sera samples analysed tested positive for IBV antibody. Using the polymerase chain reaction method, the S1 glycoprotein gene was also not detected in any of the tracheal and cloacal swabs. The overall prevalence of IBV in guinea fowl by both serology and PCR was 0% (Figure 2).

### 3.2. IBV in Local Chickens

In local chickens, none of the cloacal and tracheal swabs was positive for the S1 glycoprotein gene by PCR. Serologically, however, IBV antibodies were detected in nearly a quarter (35) of the sera samples. The seroprevalence of IBV in the local chickens was determined to be 21.2% (Figure 2). IBV prevalence among local chickens differed between the two communities. Local chickens from Frafraha were more seropositive for IBV than local chickens from Abokobi. The prevalence of IBV in local chickens from Frafraha was 30% and that of Abokobi was determined to be 7.7%. The observed difference was significant (*X*^2^= 7.424, p value= 0.006). Of the 219 sera analysed from both poultry species 35 tested positive to IBV and 184 tested negative to IBV. The overall seroprevalence of IBV in the bird species in the two (2) communities was 16.0%.

The local chicken and guinea fowl play an important role in the provision of cheap animal protein for many households in Africa. They are a source of lean meat and eggs and contribute to the reduction of protein malnutrition of the family [7, 10]. A substantial proportion of egg consumers in southern Ghana prefer eggs from local chickens to that of commercial chickens, as they are perceived to be more attractive and nutritious [21]. These local poultry additionally serve as a safety net of petty cash for the household. In certain communities, they play vital socioeconomic roles and are used as gifts and dowries for the payment of bride price. They are also used in traditional ceremonies, festivals, and rituals, honouring guests, controlling pests, and alerting owners of the presence of dangerous animals in the neighbourhood [6, 7] (Mburu and Ondwasi 2005; Avornyo et al., 2013; Dei et al., 2014).

In our study area, we observed that local chickens and guinea fowls were kept by households in both communities but guinea fowls were more likely to be found in the Abokobi community than the Frafraha community (Table 1) and the likelihood of finding local chickens was higher in Frafraha than Abokobi although not significant (can we ascribe any reason for this observation?). In general, the numbers of guinea fowls encountered in both communities were low compared to what pertains in the Northern part of the country where guinea fowls rather than local chickens are often kept by households [6]. All the local chickens and guinea fowls in the study area were raised under free-range corroborating reports from other parts of the world that local chickens and guinea fowls are mostly kept on extensive system by households [6, 10].

All the local chickens from Abokobi had received at least one dose of I2 vaccine against Newcastle disease virus but none of the chickens from Frafraha had been vaccinated. The vaccination follows efforts by the Ministry of Food and Agriculture's to assist households who keep village chickens in reducing losses due to Newcastle disease. The reasons for local chickens in Frafraha not being vaccinated with I2 were not pursued in this study. All the birds from both communities had not been vaccinated against IBV. Vaccination of both local and commercial chickens against IBV is not part of the scheduled vaccination of the Ghana Veterinary Services Directorate [6]. The detection of antibodies against IBV in commercial chickens in the Ga-East district of the Greater Accra region as reported by Ayim-Akonor et al. [17] shows the importance of including IBV vaccination in the schedule.

The youngest poultry sampled was more than 3 months old and therefore maternal antibodies against IBV if present would have waned before sampling. The detection of IBV antibodies in our test sera from local chickens is therefore an indication of exposure to field IBV pathogens. Our study shows that local chickens in the two communities have been exposed to IBV. This confirms reports that the virus circulates in the Ga-East district in which both communities are located [17]. The circulation of IBV in local chickens in the district calls for further investigation to ensure that they are not and will not become reservoirs for continual transmission of the virus.

IBV can be detected in the trachea during the acute stage of an infection. The virus however persists in the caecal tonsils where it is shed for several weeks in faeces after an infection (de Wit et al., 2014). Our sampled birds showed no respiratory symptoms, thereby decreasing our chances of detecting viral RNA in the sampled trachea. Viral shedding time via faeces could have also elapsed during our sampling time because viral RNA was not detected in the cloacal swabs of all the samples including sera positive local chickens.

Despite the circulation of IBV in the local chickens in the district and their intermingling with free-range birds, guinea fowls tested negative to IBV by both serology and PCR. The nondetection of IBV in guinea fowls could be attributed to a number of reasons. First of all, apart from chickens, the only other avian species with confirmed IBV infection are commercially reared pheasants [22, 23]. However, with antibodies to IBV having been reported in other bird species, including turkeys and quails [24, 25], we considered that it is worth investigating the possibility of chicken-cohabiting guinea fowls being infected. Additionally, the fewer guinea fowls sampled could lead us to miss any few positives within the population if the prevalence was low to begin with. It is also not clear whether the age range of the guinea fowls was significant in the negative results obtained. To our knowledge, there was no literature to indicate that it would be so. Protection from maternal antibodies would have waned by the age we sampled. However, it is also possible that because they were young, they may not have yet lived long enough to have been exposed. It appears, however, that guinea fowls are not susceptible to IBV infections but to other types of coronaviruses that are antigenically and or genetically related to IBV as reported elsewhere [26–28].

The IBV prevalence in local chickens reported here (21.2%) is lower compared to that reported in other West African countries by Emekpe et al. [29] and Adebiyi and Fagbohun [30] both from Nigeria and Kouakou et al. [31] from Côte d'Ivoire. This wide variation could result from the smaller number of samples used in this study. The lower prevalence in free-range chickens observed could also be attributed to the absence of IBV vaccine usage in commercial poultry in the country. Vaccinations with suitable IBV serotype(s) remain the practical method of controlling IBV infections in poultry population [16, 32]. Both inactivated and live attenuated IBV vaccines are used in the commercial poultry industry in Nigeria and Côte d'Ivoire to control the disease. This introduces vaccine viral strains into the environment which could be transmitted horizontally to the local chickens via aerosol, feed, or water as they scavenge for food in the neighbourhood of these commercial birds [33]. Thus local chickens from these countries could have been exposed to field IBV and/or vaccine strains against which antibodies would have been developed. Hence the resultant high IBV prevalence recorded in the studies in those countries. We did not observe a significant association between age and IBV seroprevalence (data not shown) but a significant difference was observed among local chickens in the 2 communities. We believe the variation could be due to differences in sample size and not environmental, host, or pathological factors.

## 4. Conclusion

Indigenous chickens in the Abokobi and Frafraha communities in the Ga-East district of the Greater Accra region of Ghana have been exposed to infectious bronchitis virus. Further studies are necessary to ascertain the source of infection and whether the indigenous chickens are susceptible not only to IBV, but also to infectious bronchitis disease.

## Figures and Tables

**Table 1:** Distribution of poultry types sampled in the two (2) communities.

**Poultry type**	**Abokobi (**%**)**	**Frafraha (**%**)**
Guinea fowl	48 (88.9)	6 (11.1)
Local chicken	65 (39.4)	100 (60.6)
Total	113 (51.6)	106 (48.4)

## Data Availability

Data is available in the manuscript. Further data will be made available upon request.
